# The ANGPTL3-4-8 model, a molecular mechanism for triglyceride trafficking

**DOI:** 10.1098/rsob.150272

**Published:** 2016-04-06

**Authors:** Ren Zhang

**Affiliations:** Center for Molecular Medicine and Genetics, School of Medicine, Wayne State University, 540 East Canfield Street, Detroit, MI 48201, USA

**Keywords:** Angptl3, Angptl4, Angptl8, lipasin, lipoprotein lipase, triglyceride

## Abstract

Lipoprotein lipase (LPL) is a rate-limiting enzyme for hydrolysing circulating triglycerides (TG) into free fatty acids that are taken up by peripheral tissues. Postprandial LPL activity rises in white adipose tissue (WAT), but declines in the heart and skeletal muscle, thereby directing circulating TG to WAT for storage; the reverse is true during fasting. However, the mechanism for the tissue-specific regulation of LPL activity during the fed–fast cycle has been elusive. Recent identification of lipasin/angiopoietin-like 8 (Angptl8), a feeding-induced hepatokine, together with Angptl3 and Angptl4, provides intriguing, yet puzzling, insights, because all the three Angptl members are LPL inhibitors, and the deficiency (overexpression) of any one causes hypotriglyceridaemia (hypertriglyceridaemia). Then, why does nature need all of the three? Our recent data that Angptl8 negatively regulates LPL activity specifically in cardiac and skeletal muscles suggest an Angptl3-4-8 model: feeding induces Angptl8, activating the Angptl8–Angptl3 pathway, which inhibits LPL in cardiac and skeletal muscles, thereby making circulating TG available for uptake by WAT, in which LPL activity is elevated owing to diminished Angptl4; the reverse is true during fasting, which suppresses Angptl8 but induces Angptl4, thereby directing TG to muscles. The model suggests a general framework for how TG trafficking is regulated.

## 1. Lipoprotein lipase

Triglycerides (TG), the main form of lipids to store and provide energy to the body, are essential to human life. To allow TG to circulate in the blood system, lipids are emulsified by proteins, forming lipoproteins. Chylomicrons and very-low-density lipoprotein (VLDL) are the two major TG-rich lipoprotein classes. Following a meal, chylomicrons are formed from dietary TG in mucosal cells within the villi of the duodenum, and reach the bloodstream through the lymphatic system. During fasting, VLDL is produced in the liver by TG synthesis, and is secreted directly into the bloodstream. These TG-rich lipoproteins transport and distribute TG to various tissues for either storage or oxidation to generate energy. In capillaries of these tissues, TG hydrolysis and uptake of the resulting fatty acids are largely dependent on a single enzyme, lipoprotein lipase (LPL) [1–5].

The discovery of LPL stemmed from a serendipitous observation made by Hahn, more than seven decades ago, that heparinized plasma cleared diet-induced lipaemia in dogs, but heparin by itself did not have this effect, indicating that heparin injection released a factor that cleared fat in the plasma [6]. As Hahn [6] noted ‘This phenomenon was so striking, even in the instances where the degree of lipemia was such that the plasma was suggestive of light cream’. This heparin-released clearing factor was later identified as LPL [7], because its activation depends on apolipoprotein C2, a component of lipoproteins, including VLDL, high-density lipoproteins (HDL) and chylomicrons [1–5].

LPL is a rate-limiting enzyme for hydrolysing TG presenting in circulating lipoproteins, generating free fatty acids that are taken up by peripheral tissues [8], including the heart [9–11], muscle [12–14] and fat [4]. LPL is abundantly expressed in the heart and skeletal muscle, which mainly depend on fatty acid oxidation for energy production, and in white adipose tissue (WAT), which stores energy by re-synthesis of TG from absorbed fatty acids [15]. In both humans and mice, deficiency of LPL results in severe hypertriglyceridaemia [16–18]. Because of the critical role that LPL plays in lipoprotein metabolism and tissue-specific substrate delivery and utilization, LPL activity is carefully orchestrated in a tissue-specific manner to meet the energy demands of various tissues at different nutritional statuses. For instance, feeding upregulates LPL activity in WAT but downregulates its activity in the heart and skeletal muscle; the reverse is true during fasting [4]. It is generally accepted that most physiological variations in LPL activity, such as during the fed–fast cycle, are determined by post-translational mechanisms involving interacting proteins, including apolipoproteins and members of angiopoietin-like protein family (Angptl) [4].

## 2. GPIHBP1

LPL hydrolyses TG in TG-rich lipoproteins on the surface of capillaries of peripheral tissues, including the heart, skeletal muscle and WAT; however, LPL is not expressed by capillary endothelial cells, but is produced by the parenchymal cells, myocytes and adipocytes [3]. Therefore, LPL must be transported across endothelial cells to the luminal surface of capillaries. An important discovery regarding LPL biology is that an endothelial cell protein glycosylphosphatidylinositol-anchored high density lipoprotein binding protein 1 (GPIHBP1) transports LPL into capillaries, where LPL remains anchored to the capillary wall by GPIHBP1 [19–21]. In Gpihbp1 knockout (KO) mice, LPL is mislocalized to the interstitial spaces surrounding myocytes and adipocytes, and the KO mice exhibit severe hypertriglyceridaemia (chylomicronaemia) [20,21]. In humans, GPIHBP1 loss-of-function mutations result in familial chylomicronaemia [22–25]. Without GPIHBP1, LPL cannot reach the capillary lumen, and TG-rich lipoproteins do not bind to the lumen of capillaries [19]. GPIHBP1 is, therefore, required for LPL to function on the capillary surface and is a key platform for the lipolytic processing of TG-rich lipoproteins [26–28].

## 3. Angptl3 and Angptl4

Angptl3 and Angptl4 are well-established inhibitors of LPL [29]. The first hint for involvement of Angptl proteins in lipid metabolism was from the study of KK/San mice, which exhibit extremely low serum TG levels. By performing positional cloning, Koishi *et al.* [30] identified a loss-of-function mutation in Angptl3 in these mice, suggesting that the low TG level is due to Angptl3 deficiency. Angptl3 is a circulating factor secreted from the liver, where it is specifically expressed [30]. Furthermore, Angptl3 overexpression, either by adenovirus infection or by recombinant protein i.v. injection, rescues the low TG phenotypes of KK/San mice, and leads to hypertriglyceridaemia in wild-type mice [30]. Consistently, deletion of Angptl3 in mice lowers serum TG and cholesterol levels [29,31].

Mechanistically, Angptl3 increases circulating TG levels by inhibiting LPL activity. In mice lacking Angptl3, the clearance rate of VLDL-TG was increased, whereas VLDL-TG synthesis or secretion was not affected [32]. Angptl3 has two functional domains, an N-terminal coiled-coil domain and a C-terminal fibrinogen-like domain. Angptl3 is proteolytically cleaved by proprotein convertases via recognition at the position 221–224 to yield the N-terminal domain, which is sufficient and necessary for LPL inhibition [33,34]. An Angptl3 monoclonal antibody binding to the N-terminal domain, consistently, lowers serum TG levels in mice and monkeys [35,36]. In Angptl3 KO mice, LPL activity as well as VLDL-TG incorporation are increased in oxidative tissues, including heart, muscle and brown fat [37].

Angptl4 was identified as a novel Angptl family member induced by fasting via the peroxisome proliferator-activated receptor (PPAR) in adipocytes [38–40]. Angptl4 is a potent LPL inhibitor [29,41], and plays an important role in regulating LPL activity under conditions of fasting and exercise [42]. Similar to the domain structure of Angptl3, Angptl4 is cleaved at the conserved proprotein convertase recognition sequence at position 161–164, RRKP, to release the N-terminal coiled-coil domain, which potently inhibits LPL [43,44]. Different mechanisms by which Angptl4 inhibits LPL have been proposed [45–48]. The N-terminal domain of ANGPTL4 irreversibly inhibits LPL activity by disrupting its dimerization, converting the enzyme into inactive monomers [47,48]. Using a cell-culture system to examine LPL complexed to GPIHBP1 on the endothelial cell surface, Chi *et al.* [46] showed that Angptl4 can bind and inactivate LPL complexed to GPIHBP1 and that inactivation of LPL by Angptl4 greatly reduces the affinity of LPL for GPIHBP1.

Mice injected with a monoclonal antibody against the Angptl4 N-terminal domain exhibit phenotypes similar to those of Angptl4-null mice, such as low plasma TG levels [35,49]. Indeed, Angptl4-null mice exhibit lower plasma TG and increased post-heparin plasma LPL activity; conversely, injection of recombinant Angptl4 or its transgenic overexpression increases plasma TG [29,41]. Angptl4 appears to inhibit LPL in an adipose-specific manner [50,51]. For instance, by cold exposure, the amount of labelled TG incorporated into WAT and BAT was altered in Angptl4 KO mice, whereas TG incorporation into muscle was comparable between KO and wild-type mice [50].

Sequence variations of ANGPTL3 and ANGPTL4 are robustly linked to lipid profiles by genome-wide association studies (GWAS). In humans, homozygotes or compound heterozygotes for loss-of-function mutations of ANGPTL3 cause familial combined hypolipidaemia, characterized by a reduction of all lipoprotein classes, such as VLDL, LDL and HDL [52,53]. The E40K substitution in ANGPTL4 is associated with lower plasma TG and HDL-C concentrations [54,55]. Re-sequencing of protein-coding regions showed that 1% of the Dallas Heart Study (DHS) population and 4% of those participants with a plasma TG in the lowest quartile have loss-of-function mutations in ANGPTL3, ANGPTL4 or ANGPTL5 [56].

## 4. Lipasin/Angptl8

The functional roles in lipid metabolism of a previously uncharacterized gene, Gm6484, were discovered and reported by multiple groups in 2012, under various names, such as RIFL [57], lipasin [58], Angptl8 [59] and betatrophin [60]. In October 2015, the HUGO gene nomenclature committee [61] assigned the official name of this gene as ANGPTL8 (human) and Angptl8 (mouse), which are adopted in the current review. Active research on Angptl8 in the past years has provided critical information on its function, mechanism of action and therapeutic potential [62,63].

We overexpressed Angptl8 in the mouse liver using adenovirus through tail vein injection, and Angptl8 overexpression led to dramatically increased serum TG levels [58]. Quagliarini *et al.* [59] found that overexpressed Angptl8 increased serum TG levels in an Angptl3-dependent manner. Mice lacking Angptl8 consistently exhibit lower TG levels owing to enhanced plasma TG clearance by having increased post-heparin LPL activity [64,65]. We recently found that Angptl8 KO mice have higher LPL activity specifically in cardiac and skeletal muscles [66]. This result suggests that Angptl8 negatively regulates LPL activity in these two tissues. Furthermore, Angptl8 is a therapeutic target because its neutralization with a monoclonal antibody (epitope being E_97_IQVEE) lowers serum TG levels [66].

Angptl8 expression is highly enriched in the liver, WAT and BAT [57–59]. Furthermore, Angptl8 expression is reduced by fasting, and is highly induced by feeding in both liver and adipose tissues [57–59]. In brown fat, Angptl8 is upregulated by cold exposure [67]. Using mice lacking different isoforms of sterol regulatory element-binding protein (Srebp), Angptl8 was shown to be induced by feeding independent of Srebp [59]. AMP-activated protein kinase was shown to suppress LXR/SREBP-1 signalling-induced Angptl8 expression in HepG2 cells [68]. Angptl8 is highly regulated during adipogenesis, and its knockdown significantly suppresses adipocyte differentiation [57]. Additionally, Angptl8 is upregulated by thyroid hormone and modulates autophagy [69].

In humans, ANGPTL8 sequence variations have been demonstrated to be associated with lipid profiles by GWAS. Three ANGPTL8 SNPs are strongly associated with lipid profiles. The first SNP, rs2278426, represents a nucleotide transition (C versus T, from CGG to TGG) that results in a non-synonymous amino acid change, from arginine (R) to tryptophan (W) at residue 59. Quagliarini *et al.* [59] found that the 59W variant is associated with lower LDL-C and HDL-C levels in various ethnic groups. Consistently, in a study composed of 4361 Mexicans, Weissglas-Volkov *et al.* found that WW homozygotes had 14% lower HDL-C than RR homozygotes. African Americans in the DHS had 15% lower LDL-C in WW homozygotes than in RR homozygotes [70]. The second SNP, rs145464906, represents a nucleotide transition (C versus T, from CAG to TAG) that results in a premature stop codon at residue 121, and therefore a truncated ANGPTL8 is generated by this SNP. The carriers of this presumably partial loss-of-function mutation with European ancestry were 10 mg dl^−1^ higher in HDL-C and 15% lower in TG levels [71]. The third SNP, rs737337, has also been found to be associated with HDL-C levels [28], and this SNP is located in the upstream region of the ANGPTL8 transcription start site [72].

Circulating levels of ANGPTL8 in human physiology and pathology have been an area of active investigation. The circulating levels of ANGPTL8 in humans were found to be decreased by overnight fasting [59] and increased 2 h following a defined meal [73]. Circulating ANGPTL8 levels were found to increase in type 2 diabetes [73–80], gestational diabetes [81–83], obese children with insulin resistance [84] and type 1 diabetes [78,85]. Nevertheless, the relationship between circulating levels of ANGPTL8 and diabetes and obesity remains inconclusive [86,87]. ANGPTL8 levels were also found to be associated with other metabolic conditions [88–96].

Therefore, overwhelming evidence from both loss- and gain-of-function studies in mice as well as human GWAS has demonstrated that Angptl8 is a feeding-induced hepatokine that is a potent regulator of lipid metabolism.

## 5. The Angptl3-4-8 model

TG are directed to WAT for storage after feeding, and to the heart and skeletal muscle for oxidation to generate energy during fasting. It is now clear that the process of TG trafficking is critically determined by LPL. After feeding, LPL activity rises in WAT but declines in muscles; conversely, during fasting, LPL activity declines in WAT but rises in muscles. Nevertheless, the mechanism for regulating tissue-specific LPL activity during the fed–fast cycle remains largely unknown.

**Figure 1.**
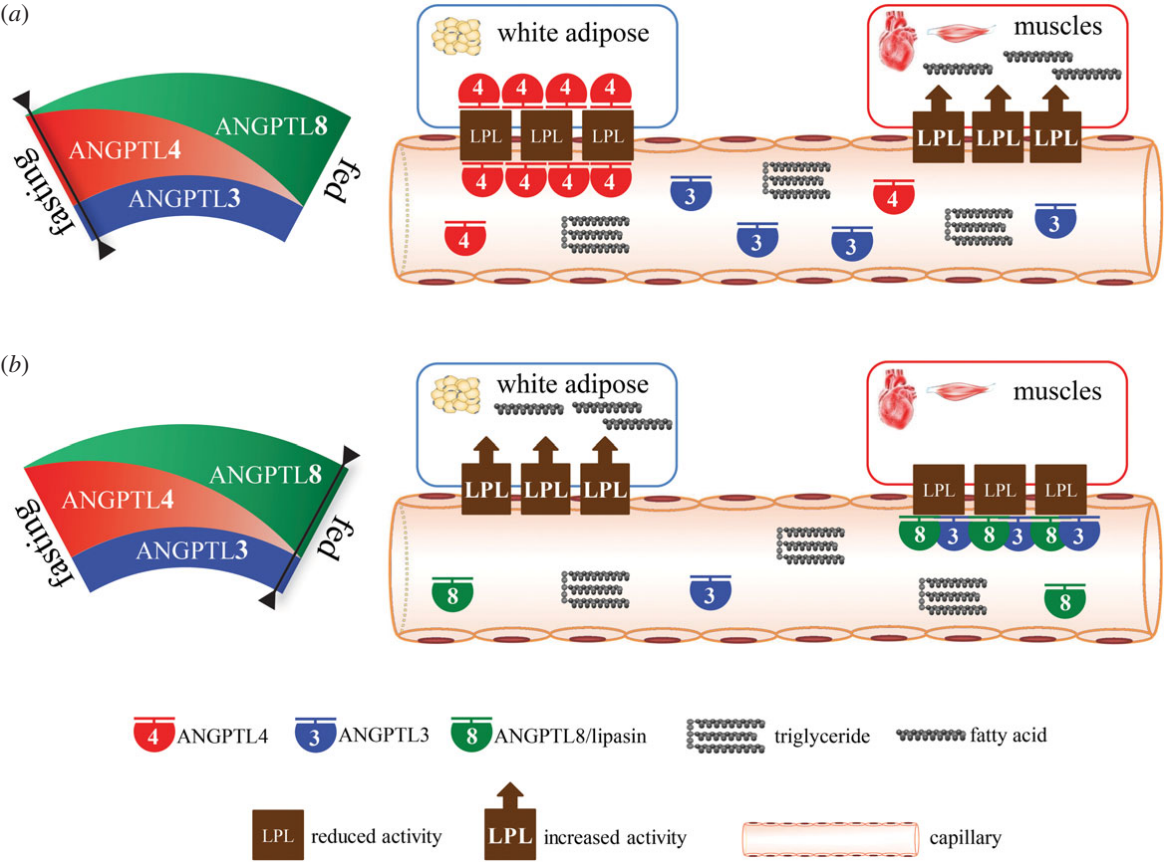
The ANGPTL3-4-8 model. ANGPTL8, ANGPTL3 and ANGPTL4 regulate triglyceride (TG) trafficking by inhibiting lipoprotein lipase, in a tissue-specific manner, under different nutritional statuses. The level of ANGPTL3 is stable, regardless of nutritional status, but it requires activation by ANGPTL8. Fasting induces ANGPTL4, which inhibits LPL in WAT to direct circulating TG to cardiac and skeletal muscles for oxidation (*a*); conversely, feeding induces ANGPTL8, activating the ANGPTL8–ANGPTL3 pathway, which inhibits LPL in cardiac and skeletal muscles to direct circulating TG to WAT for storage (*b*).

The discoveries of Angptl3 and Angptl4 have offered significant insights into this process, as both are potent LPL inhibitors. However, based on Angptl3 and Angptl4 only, the LPL regulation among WAT, the heart and skeletal muscle cannot be explained. The discovery of Angptl8 seems to complete the player set for LPL regulation, but it is puzzling that all the three Angptl members are LPL inhibitors, and that deficiency (overexpression) of any one of them results in hypotriglyceridaemia (hypertriglyceridaemia). Then why does nature need all of the three Angptl members for regulating LPL activity? Our finding that Angptl8 negatively regulates LPL activity specifically in the heart and skeletal muscle immediately suggested a model by which TG trafficking regulation is explained by Angptl3, Angptl4 and Angptl8 (Angptl3-4-8 model; figure 1) [66].

According to this model, Angptl8 activates Angptl3, in an endocrine manner, to inhibit the activity of LPL in the heart and skeletal muscle, whereas Angptl4, involving intracellular and circulating species, inhibits LPL activity in WAT. Fasting upregulates Angptl4 but downregulates Angptl8, and consequently LPL activity in WAT is reduced but in muscles is increased, and therefore TG are directed to muscles for oxidation. Conversely, food intake downregulates Angptl4 but upregulates Angptl8, and consequently LPL activity in WAT is increased but in muscles is reduced, thereby directing circulating TG to WAT for storage (figure 1) [66].

Several provocative findings should be noted. In 1964, Eagle & Robinson [97] demonstrated that in WAT, blocking the transcription using actinomycin increases LPL activity during fasting. Consistently, Olivecrona and co-workers [98] showed that expression of a gene needs to turn on to downregulate adipose LPL activity. Now, it has become clear that this hypothesized fasting-induced protein that inhibits WAT LPL is Angptl4. By studying a wide array of mouse strains, Ben-Zeev *et al.* [99] suggested that separate genes regulate LPL activity in adipose tissue and in the heart. Importantly, Olivecrona and co-workers [100] found that, similar to adipose tissue, a transcription-dependent mechanism is involved in modulating heart LPL activity. Following actinomycin D injection, postprandial LPL activity in the heart was increased. Therefore, they proposed that feeding induced a protein that inhibits postprandial cardiac LPL activity [100]. It is likely that this hypothesized feeding-induced protein is Angptl8.

## 6. Angptl8 and Angptl3 function in the same pathway

Current evidence supports a notion that Angptl8 and Angptl3 function in the same pathway, that is, Angptl8 inhibits LPL, in an Angptl3-dependent manner, in cardiac and skeletal muscles, whereas Angptl3, although being abundant in the circulation regardless of nutritional status, needs to be activated by Angptl8, which is induced by feeding. By jointly considering the Angptl3-4-8 model and phenotypes of mice deficient in Angptl8 or Angptl3, we can obtain further insights into the relationship between the two Angptl members.

Angptl8 KO mice exhibited higher LPL activity in cardiac and skeletal muscles in the fed state, suggesting that Angptl8 is required for LPL inhibition in these tissues [66]. This result was obtained in mice with abundant Angptl3, suggesting that in the absence of Angptl8, Angptl3 does not effectively suppress LPL in these tissues. In other words, Angptl3 requires Angptl8 to be functionally active to inhibit LPL in cardiac and skeletal muscles.

Angptl3 KO mice exhibited higher LPL activity in cardiac and skeletal muscle in the fed state [37], suggesting that Angptl3 is required for LPL inhibition in these tissues. This result was obtained in the fed state, that is, Angptl8 was abundant, suggesting that Angptl8 required Angptl3 to inhibit muscle LPL. Consistently, hepatic Angptl8 overexpression in mice dramatically increased serum TG levels [58], but this increase was abolished in the Angptl3 KO mice [59].

It was shown that Angptl8 interacts with Angptl3, and enhances Angptl3 cleavage, releasing the N-terminal domain, which potently inhibits LPL [59]. This result leads to multiple possibilities for the mechanisms of how Angptl8 and Angptl3 function. One possibility is that Angptl8 enhances Angptl3 cleavage, releasing the N-terminal domain, which in turn targets muscle LPL, but Angptl8 itself remains in the circulation. Another possibility is that the two proteins form a complex that translocates to muscle capillaries to inhibit LPL. The latter seems more likely for the following reasons. Angptl8 KO mice did not exhibit reduced levels of the Angptl3 N-terminal domain [65], and thus Angptl8 is not required for Angptl3 cleavage. Furthermore, in mice with Angptl8 overexpression circulating Angptl3 levels were reduced [59], supporting the notion that exogenous Angptl8 formed complexes with Angptl3, which, in turn, translocated into the capillaries in the heart and skeletal muscle, resulting in lowered levels of circulating Angptl3.

Both Angptl8 and Angptl3 are secreted by the liver into the circulation, and are not expressed in the heart and skeletal muscle, and thus are likely to work in an endocrine manner. Taken together, these results strongly suggest that Angptl8, induced by feeding, binds and activates Angptl3 to inhibit LPL in cardiac and skeletal muscles, in an endocrine manner.

## 7. Explanation of triglyceride levels in mice with altered expression of Angptl8, Angptl3 or Angptl4 by the Angptl3-4-8 model

**Figure 2.**
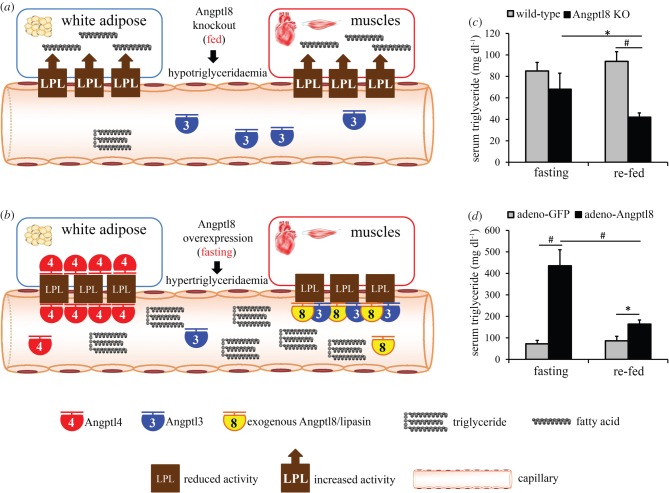
Changes in triglyceride levels in Angptl8-deficient or overexpressing mice explained by the Angptl3-4-8 model. (*a*) In the fed state, Angptl8-null mice have high LPL activity in both WAT and muscles, resulting in lower circulating TG levels. (*b*) Conversely, in the fasting state, Angptl8-overexpressing mice have low LPL activity in both WAT and muscles, resulting in dramatically higher circulating TG levels. (*c,d*) TG levels in Angptl8 KO (*c*) and overexpressing (*d*) mice. Mice were fasted for 24 h or re-fed for 4 h following the fast. Panels (*c,d*) are reproduced from data in figure 3 of [66] with permission of Scientific Reports. Data are presented as mean ± s.e.m. *N* = 6–8 per group. KO, knockout; WAT, white adipose tissue. *p < 0.05; ^#^*p* < 0.01.

A striking phenotype in Angptl8 KO mice is that refeeding decreases serum TG levels [65,66] (figure 2*c*). This striking phenotype is nicely explained by the model. According to the Angptl3-4-8 model, in Angptl8 KO mice, LPL activity in the heart and skeletal muscle remains active in both fasting and fed states. However, in the fasting state abundant Angptl4 inhibits WAT LPL, while following refeeding Angptl4 is diminished, resulting in higher WAT LPL activity, enhanced WAT fatty acid uptake, enhanced circulating TG clearance and thus lower serum TG levels.

In the fed state, Angptl8 KO mice also have low levels of Angptl4, resulting in higher activity of LPL in both WAT and muscles, and therefore circulating TG are effectively hydrolysed and taken up by both WAT and muscles, leading to hypotriglyceridaemia [65,66] (figure 2*a,c*). In the fasting state, because Angptl4 is induced, WAT LPL inhibition is retained, and therefore circulating TG levels showed no significant difference from those of wild-type mice (table 1 and figure 2*c*). In the case of Angptl8 overexpression, in the fed state, because LPL activity in WAT is still high in the absence of Angptl4, the elevation of circulating TG is modest. In the fasting state, however, LPL in both WAT and muscles is inhibited, resulting in striking elevation of circulating TG (figure 2*b,d*).

**Table 1.** Serum triglyceride levels and LPL activity in mice with altered Angptl8 expression [66]. ↑, increased; ↓, decreased; −, unchanged; **^**, slightly increased.

	serum triglycerides		LPL activity in cardiac and skeletal muscles		LPL activity in WAT	
Angptl8 levels	fed	fasting	fed	fasting	fed	fasting
deficiency^a^	**↓**	**—**	**↑**	**—**	**—**	**—**
overexpression^b^	**^**	**↑**	**—**	**↓**^c^	**—**	**—**

^a^Angptl8 knockout.

^b^Mice with adenovirus-Angptl8 injection.

^c^Only in the heart.

In the Angptl3 KO mice, in the fed state, induced Angptl8 cannot inhibit muscle LPL because this inhibition is Angptl3-dependent, and therefore LPL activity is high in both WAT and muscles, resulting in hypotriglyceridaemia, whereas in the fasting state, inhibition of LPL by Angptl4 is retained, and therefore the hypotriglyceridaemia phenotype is relatively modest [37].

**Figure 3.**
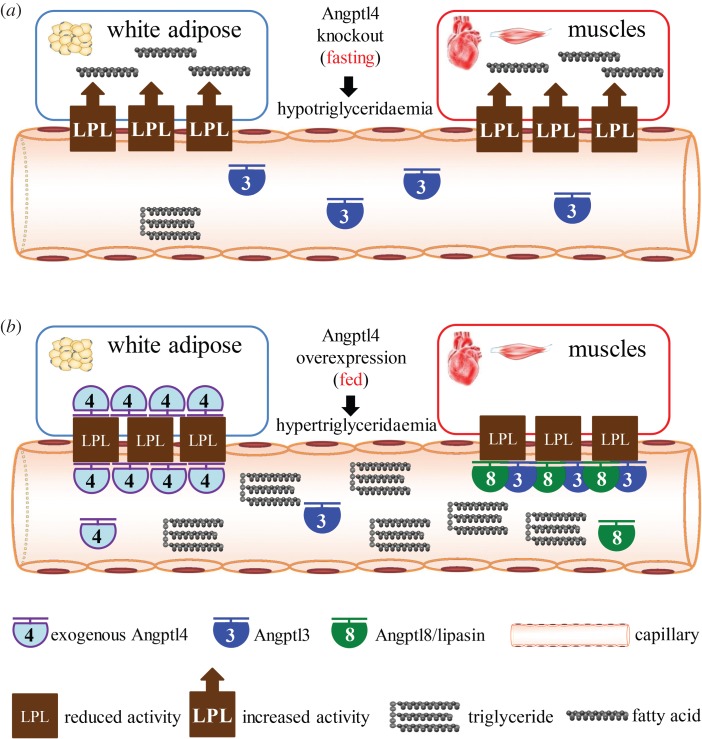
Changes in triglyceride levels in Angptl4 deficient or overexpressing mice explained by the Angptl3-4-8 model. (*a*) In the fasting state, Angptl4-null mice have high LPL activity in both WAT and muscles, resulting in lower circulating TG levels. (*b*) Conversely, in the fed state, Angptl4 overexpressing mice have low LPL activity in both WAT and muscles, resulting in dramatically higher circulating TG levels.

In Angptl4 KO mice, in the fasting state, LPL activity in both WAT and muscle is high, resulting in hypotriglyceridaemia. In the fed state, inhibition of LPL in muscles is retained, and therefore the low TG phenotype is relatively modest compared with that in the fasting state [29,51]. In the case of Angptl4 overexpression, in the fed state, LPL activity in both WAT and muscles is low, and therefore hypertriglyceridaemia results, whereas in the fasting state, we hypothesize that the hypertriglyceridaemia would be relatively modest because the low Angptl8 and associated high LPL activity in muscles would result in muscle uptake of TG-derived fatty acids (figure 3).

## 8. Limitations of the Angptl3-4-8 model

The Angptl3-4-8 model, obviously, is not a perfect one. First, the model does not explain the functional role of Angptl8 in adipose tissues. In WAT, Angptl8 expression is strongly induced by feeding [57]. However, WAT LPL activity is upregulated by feeding as well [51]. In Angptl8 KO mice, we found that WAT LPL activity was not affected [66]. Furthermore, in Angptl8 KO mice, uptake of VLDL-TG by WAT was decreased [65]. These results are inconsistent with a role of Angptl8 in inhibiting WAT LPL. Therefore, Angptl8 in WAT may be involved in functions independent of LPL, such as adipogenesis [57]. Likewise, in BAT, LPL activity is upregulated by cold exposure, whereas Angptl8 is also upregulated [67]. Therefore, we hypothesize that Angptl8 may have LPL-independent functions in both WAT and BAT. Although it has been well established that Angptl8 is a circulating factor secreted from the liver, it should be noted that, thus far, there has been no evidence to suggest that Angptl8 is also secreted from adipose tissues. It is likely that Angptl8 in WAT and BAT functions in a non-endocrine manner.

Angptl3 KO mice exhibited elevated LPL activity in WAT, in addition to increased postprandial LPL activity in oxidative tissues [37]. This result suggests that Angptl3 may also play a role in inhibiting LPL in WAT. Because the Angptl3 level is not nutritionally regulated, it is possible that Angptl3 is a general LPL inhibitor needed by both Angptl4 and Angptl8. However, currently, there has been no evidence showing Angptl4 and Angptl3 can interact to regulate LPL.

Angptl4 has a relatively wide expression pattern, including expression in skeletal muscle and the heart [4]. In Angptl4 KO mice, however, in response to cold exposure, TG incorporation was altered specifically in adipose tissues, but not in muscles [50], consistently with the Angptl3-4-8 model. The contributions of intracellular versus circulating Angptl4, as well as Angptl4 expressed in non-adipose tissues, to LPL inhibition are unclear.

To fully prove the model, it is necessary to examine tissue-specific uptake of labelled TG in Angptl8 and Angptl4 KO mice during the fed–fast cycle. Future models will probably incorporate functional roles of Angptl8 and Angptl3 in WAT, Angptl4 in non-WAT tissues and other factors involved in LPL regulation, such as apoA-V and apoC-III [101].

## 9. Perspective

Since the discovery of Angptl8 in 2012 [57–59], significant progress has been made in elucidating the functional role, mechanism of action and therapeutic potential of this protein [62,63]. With the Angptl8 discovery, a model of how TG trafficking is coordinated at various nutritional states emerged [66]. Although the Angptl3-4-8 model still leaves much room to be improved, it provides a framework that links Angptl3, Angptl4, Angptl8 and LPL. Below are some outstanding questions to address.

### 9.1. Mechanism of action

The mechanism of LPL inhibition by Angptl8 remains obscure. One possible mechanism of action is that Angptl8 enhances cleavage of Angptl3, releasing the N-terminal domain, which, in turn, inhibits LPL. Another possibility is that Angptl8 binds to Angptl3, forming a complex that inhibits LPL in the heart and skeletal muscle. The two scenarios are distinct in that in the former Angptl8 does not translocate to the heart and skeletal muscle, but stays in the circulation only, whereas in the latter Angptl8 is physically located in these tissues and has interactions with Angptl3 and/or LPL. As discussed in §6, the second scenario seems more likely; however, direct experimental evidence, such as immunohistostaining showing the presence of Angptl8 and Angptl3 on the surface of capillaries in the heart and skeletal muscle, is lacking.

A related question is: what is the mechanism ensuring tissue specificity of LPL inhibition by the Angptl8–Angptl3 pathway in cardiac and skeletal muscles, and by Angptl4 in WAT? Because Angptl8 and Angptl3 are likely to function in an endocrine manner, it is almost certain that they encounter LPL in capillaries of peripheral tissues, in which LPL is anchored by GPIHBP1 to the capillary endothelial lumen. GPIHBP1 has been shown to play an important role in mediating inhibition of Angptl proteins on LPL [46,102]. However, the roles of GPIHBP1 on Angptl8- and Angptl3-mediated inhibition of LPL and on establishing their functional tissue specificity remain unknown.

### 9.2. Transcriptional regulation

Angptl8 is strongly induced by feeding and suppressed by fasting. As we pointed out, levels of Angptl8 and Angptl4 show opposite changes in response to various stimuli, such as fasting, feeding, insulin resistance and cold exposure [62,63,67]. Likewise, a recent report showed that glucagon receptor antagonists upregulate Angptl4, while downregulating Angptl8, and that the induction of adipose Angptl4 specifically promotes pancreatic α-cell proliferation [103]. This reciprocal regulation is critical to balance the abundance of Angptl8 versus Angptl4 to regulate cellular processes in different physiological and pathological settings. Because Angptl8 is expressed in liver and adipose tissues, it is likely that transcription factors mediating Angptl8 transcription are different in the two tissues. The carbohydrate-responsive element-binding protein (ChREBP), a glucose-responsive transcription factor [73], and PPARγ [57] were suggested to mediate Angptl8 transcription. However, the identity of transcription factors and their binding sites in the Angptl8 promoter region have not been clearly delineated. Another question is that Angptl8 protein during fasting must be degraded quickly, but the degradation pathway and its regulation remain elusive. Elucidation of the identity of transcription factors mediating Angptl8 transcription is critical in understanding the nutritional regulation of Angptl8, as well as the reciprocal regulation of Angptl8 versus Angptl4.

### 9.3. Functions in adipose tissues

Angptl8 is strongly upregulated in WAT following food intake [57,58]. Angptl8 is abundant in mouse BAT, and is highly upregulated by cold exposure [67]. In both cases, LPL activity is increased, and therefore it is likely that Angptl8 has LPL-independent functions in adipose tissues. Angptl8 is upregulated during adipocyte differentiation, and its knockdown impairs adipogenesis in 3T3-L1 cells [57]. However, the functional roles of Angptl8 in WAT and BAT *in vivo* remain unclear.

### 9.4. Human pathology

Accumulating evidence suggests that circulating ANGPTL8 is elevated in diabetes [73–85,104]. Diabetes is often associated with diabetic dyslipidaemia, characterized by hypertriglyceridaemia, lower HDL-C and postprandial lipaemia [105]. The direct implication of the ANGPTL3-4-8 model is that when ANGPTL8 is increased, LPL activity will be suppressed in the heart and skeletal muscle, resulting in accumulation of TG in the circulation (hypertriglyceridaemia). Then, is ANGPTL8 a contributing factor in causing diabetic dyslipidaemia? We have shown that an Angptl8 monoclonal antibody lowers serum TG in mice. Can ANGPTL8 inhibition be a therapeutic approach to treat diabetic dyslipidaemia?

Homozygous or compound heterozygous loss-of-function mutations in ANGPTL3 and ANGPTL4 have been identified in humans, providing valuable information on human pathology [56]. However, homozygous loss-of-function mutations have not been found in ANGPTL8. Searching the ExAC database [106], which is based on exome sequencing results of more than 60 000 human subjects, revealed no ANGPTL8 homozygous null mutations. According to phenotypes of Angptl8 KO mice, it is unlikely that null mutations are lethal. Therefore, with the expansion of exome databases, identification of homozygous null mutations of ANGPTL8 is possible, and will provide critical information on the pathophysiology of its functions in humans.

## 10. Concluding remarks

The partitioning of TG to specific tissues according to nutritional states is a fundamental biological process, and the elucidation of the molecular mechanism of TG trafficking will have profound implications for understanding metabolic disease. The ANGPTL3-4-8 model suggests that the three Angptl members are a key in balancing the partitioning of circulating TG between WAT and oxidative tissues. Breaking this balance may lead to obesity, lipotoxicity or hypertriglyceridaemia, representing excess TG in WAT, non-adipose tissues and plasma, respectively.

Inhibition of each of the three Angptl members, based on small molecular inhibitors or monoclonal antibodies, has tremendous therapeutic potential in treating dyslipidaemia. Of note, clinical trials evaluating an ANGPTL3 monoclonal antibody are ongoing (trial https://trialbulletin.com/lib/entry/ct-02265952; trial https://trialbulletin.com/lib/entry/ct-01749878; trial https://trialbulletin.com/lib/entry/ct-02107872). The ANGPTL3-4-8 model suggests that the key to the concept of LPL-based therapeutic strategy is the balance. The goal should not be to inhibit, e.g., ANGPTL3 to the maximum extent, which may lead to lipotoxicity, but to reduce its activity to a specific level, so that abnormal TG trafficking associated with pathological conditions is corrected.

Since the discovery of LPL seven decades ago, significant progress has been made in elucidating the functional role of LPL in regulating TG trafficking. Recent discovery of lipasin/ANGPTL8 results in an ANGPTL3-4-8 model, which provides a molecular mechanism by which tissue-specific LPL activity is regulated during the fed–fast cycle. Specifically, feeding induces ANGPTL8, activating the ANGPTL8–ANGPTL3 pathway, which inhibits LPL in cardiac and skeletal muscles, thereby making circulating TG available for uptake by WAT, in which LPL activity is elevated owing to diminished ANGPTL4; the reverse is true during fasting, which suppresses ANGPTL8 but induces ANGPTL4, thereby directing circulating TG to muscles. The model may provide significant insights into the understanding of TG metabolism and metabolic disease.
